# Quasi-maximum exponential likelihood estimator and portmanteau test of double $\operatorname{AR}(p)$ model based on $\operatorname{Laplace}(a,b)$

**DOI:** 10.1186/s13660-018-1769-9

**Published:** 2018-09-11

**Authors:** Haiyan Xuan, Lixin Song, Un Cig Ji, Yan Sun, Tianjiao Dai

**Affiliations:** 1 School of Mathematical Sciences, Dalian University of Technology, Dalian, P.R. China; 2 School of Economics and Management, Lanzhou University of Technology, Lanzhou, P.R. China; 3 College of Science, Department of Mathermatics, Research Institute of Mathematical Finance, Chungbuk National University, Cheongju, Republic of Korea; 4 College of Science, Lanzhou University of Technology, Lanzhou, P.R. China

**Keywords:** 62M10, 91G70, Double $\operatorname{AR}(p)$ model, Quasi-maximum exponential likelihood estimator, Portmanteau test, Autocorrelations

## Abstract

The paper studies the estimation and the portmanteau test for double $\operatorname{AR}(p)$ model with $\operatorname{Laplace}(a,b)$ distribution. The double $\operatorname{AR}(p)$ model is investigated to propose firstly the quasi-maximum exponential likelihood estimator, design a portmanteau test of double $\operatorname{AR}(p)$ on the basis of autocorrelation function, and then establish some asymptotic results. Finally, an empirical study shows that the estimation and the portmanteau test obtained in this paper are very feasible and more effective.

## Introduction

In 1982, Engle proposed the ARCH model, and used it to analyze the volatility clustering of the inflation index. Weiss [1] considered a model called double $\operatorname{AR}(p)$ as an extension of the ARCH model. After parameter estimation and test he showed that there are various available test means to be used to test this model. The double $\operatorname{AR}(p)$ model is an $\operatorname{AR}(p)$ model with conditional heteroscedasticity. Then Francq and Zakoian gave an example of weak ARMA model in 1998 and 2000, respctively. Ling [2] applied quasi-maximum likelihood estimation (QMLE) to give a parameter estimation, then used two ways to test the stationary of double $\operatorname{AR}(1)$ model under weak conditions, finally presented an empirical study. Chan and Peng [3] presented a locally weighted least absolute deviation estimation for the double $\operatorname{AR}(1)$ model and established its asymptotic theory. Wang et al. [4] studied the heteroscedastic mixture double AR model to simulate the nonlinear time series, and proposed some stability conditions of the model. Ling and Li [5] performed the diagnostic tests for non-stationary double $\operatorname{AR}(1)$ model. Zhu and Ling [6] proposed the quasi-maximum exponential likelihood estimator for the double $\operatorname{AR}(p)$ model, and made a comparison with the weighted least squares under the finite sample condition.

The research of a diagnostic test, often accompanied by the development of a model, plays an important role in the research of the model. McLeod and Li [7] presented a diagnostic test using squared-residual autocorrelation function. Dufour and Roy [8] gave a nonparametric portmanteau test. Monti [9] proposed a portmanteau test based on residual partial autocorrelation function. Wong and Li [10] made the portmanteau test for multivariate conditional heteroscedasticity model. Francq et al. [11] proposed a diagnosis test method for weak ARMA model. Kwan et al. [12] studied the portmanteau test under the condition of finite sample. Francq [13] aiming at autoregressive models with uncorrelated but non-independent errors made the multivariate portmanteau test. And then Mainassara [14] also made a multivariate portmanteau test for structural VARMA models with uncorrelated but non-independent error terms. Kwan et al. [15] defined two portmanteau tests based on residual autocorrelation function and square residual autocorrelation function, respectively. Fisher and Gallagher [16] proposed a new weighted portmanteau statistic for the goodness of fit for time series. Zhu and Ling [17] presented a Ljung–Box portmanteau test based on symbolic function in order to test the properties of ARMA model with fat-tailed noise. Zhu [18] used the random weighting method to make a bootstrap portmanteau test on the basis of residual autocorrelation function and residual partial autocorrelation function of weak ARMA model. Recently, Xuan [19] made a portmanteau test aiming at ARFIMA–GARCH model. Stefanos [20] studied time-varying parameter regression models with stochastic volatility and made a semiparametric Bayesian inference.

The structure of this paper is as follows. The second part focuses on the parameter estimation method and the portmanteau test statistic of double $\operatorname{AR}(p)$ model derived from this method. The quasi-maximum exponential likelihood estimator and portmanteau test statistic based on residual autocorrelation function will be given in this section. In the third part, there is an empirical study of CSI 800 which applies the portmanteau test to check the double $\operatorname{AR}(p)$ model. Conclusions are given in the final section.

## Quasi-maximum exponential likelihood estimator and portmanteau test

In this section, the double $\operatorname{AR}(p)$ model with $\operatorname{Laplace}(a,b)$ distribution will be investigated to propose the quasi-maximum exponential likelihood estimator, establish some asymptotic results, and design the portmanteau test based on autocorrelation function.

### Quasi-maximum exponential likelihood estimator based on $\operatorname{Laplace}(a,b)$

Consider the double $\operatorname{AR}(p)$ model

1 $$ \begin{aligned} &y_{t} = \sum _{i = 1}^{p} \phi_{i}y_{t - i} + \eta_{t}\sqrt{\omega + \sum_{i = 1}^{p} \alpha_{i}y_{t - i}^{2}}, \\ &\varepsilon_{t} = \eta_{t}\sqrt{h_{t}},\quad h_{t} = \omega + \sum_{i = 1} ^{p} \alpha_{i}y_{t - i}^{2}, \end{aligned} $$

where $\phi_{i} \in \mathbb{\mathbf{R}}$, $\omega \ge 0$, $\alpha_{i} \ge 0$, $i = 1, \ldots,p$, $\eta_{t}$, are independent and identically distributed white noise sequences, $y_{s}$ is independent of $\{ \eta_{t}:t \ge 1\}$ for $s \le 0$, and the conditional variance of $y_{t}$ is $h_{t} = \sigma_{t}^{2} = \operatorname{var}(y _{t}| \mathcal{F}_{t - 1} ) = E\eta_{t}^{2}( \omega + \sum_{i = 1}^{p} \alpha_{i}y_{t - i}^{2} )$, where $E\eta_{t}^{2} < \infty $. In practice, the estimated value of the intercept *ω* is very small and can be considered as infinitely approaching to zero.

Let $\theta = (r',\delta ')'$ be the unknown parameters of the model, and the true value is $\theta_{0} = (r'_{0},\delta '_{0})$, $r = (\phi _{1},\phi_{2}, \ldots,\phi_{p})'$, $\delta = (\omega,\alpha_{1},\alpha _{2}, \ldots,\alpha_{p})'$. Define the parameter space $\Theta = \Theta_{r} \times \Theta_{\delta } $, and $\Theta_{r} \in \mathbb{R} ^{p}$, $\Theta_{\delta } \in \mathbb{R}_{0}^{p + 1}$, $\mathbb{R} = ( - \infty, + \infty)$, $\mathbb{R}_{0} = [0,\infty)$.

Let *X* obey $\operatorname{Laplace}(a,b)$ distribution, where *a* is a positional parameter and *b* is a scale parameter. After the transformation $Y = \frac{X - a}{b}$ is drawn, *Y* follows the $\operatorname{Laplace}(0,1)$ distribution. Therefore, we only discuss the situation of $\operatorname{Laplace}(0,1)$ distribution as follows. In order to carry out the calculation successfully, we also need the following three assumptions.

#### Assumption 1

Θ *is a compact set*, $\theta_{0}$ *is the inner point of* Θ. $\underline{\omega} \le \omega \le \bar{ \omega } $ *and* $\underline{\alpha }_{i} \le \alpha_{i} \le \bar{ \alpha }_{i}$ ($i = 1, \ldots,p$), $\underline{\omega }$, *ω̄*, $\underline{ \alpha }_{i}$, $\bar{\alpha }_{i}$ ($i = 1, \ldots,p$) *are positive constants*.

#### Assumption 2

*For* $l > 0$ *and* $E \vert y_{t} \vert ^{l} < \infty,\{ y_{t}:t = 1 - p, \ldots,0,1,2, \ldots \}$ *is a strictly stationary and ergodic sequence*.

#### Assumption 3

*In the situation of* $E\eta_{t}^{2} < \infty $, *the median of* $\eta_{t}$ *is zero*, *and it has a bounded continuous density function* $f(x)$ *in* $\mathbb{R}$ *which satisfies the range of density function* $(0, + \infty )$.

Based on the conditions of the three assumptions, in what follows we will derive asymptotic distribution of the estimators.

When $\eta_{t}$ obeys the $\operatorname{Laplace}(0,1)$ distribution, the log-likelihood function can be expressed as

$$L_{n}(\theta) = \frac{1}{n}\sum_{t = p + 1}^{n} l_{t}(\theta), $$

where

$$l_{t}(\theta) = \log \sqrt{h_{t}(\delta)} + \frac{ \vert \varepsilon_{t}(r) \vert }{\sqrt{h_{t}(\delta)}}. $$

Let

$$\hat{\theta }_{n} = \arg \min_{\Theta } L_{n}( \theta). $$

Then $\hat{\theta }_{n}$ is called the quasi-maximum exponential likelihood estimator of $\theta_{0}$. It follows from Assumptions 1–3 that $\hat{\theta }_{n}$ is obtained immediately, and the asymptotic properties of the estimators are derived as follows.

#### Theorem 1

*If Assumptions* 1*–*3 *hold*, *then*

$$\hat{\theta }_{n} \to \theta_{0} \quad \textit{a.s.},\quad \textit{as } n \to \infty. $$

It is easy to obtain the proof of Theorem 1 by using compact set theory, Markov theorem, and ergodicity theorem.

### Portmanteau test based on autocorrelation function

Let $\hat{\varepsilon }_{t} = \varepsilon_{t}(\hat{\theta }_{n})$ be double $\operatorname{AR}(p)$ model’s residual. Then the residual autocorrelation function of lag *k* is

$$\hat{\rho }_{k} = \frac{\sum_{t = 1}^{n - k} \hat{\varepsilon }_{t} \hat{\varepsilon }_{t + k}}{\sum_{t = 1}^{n} \hat{\varepsilon }_{t} ^{2}}. $$

From the definition of the autocorrelation function, we can get the sample covariance function

$$\hat{\zeta }_{k} = \frac{1}{n}\sum _{t = 1}^{n - k} \hat{\varepsilon } _{t}\hat{ \varepsilon }_{t + k}. $$

Therefore, the autocorrelation function of *k*-order can be simplified as $\hat{\rho }_{k} = \frac{\hat{\zeta }_{k}}{\hat{\zeta }_{0}}$. At the same time, let

$$\hat{\boldsymbol{\zeta }}_{k} = (\hat{\zeta }_{1},\hat{ \zeta }_{2}, \ldots,\hat{\zeta }_{k}),\qquad\hat{\boldsymbol{ \rho }}_{k} = (\hat{\rho } _{1},\hat{\rho }_{2}, \ldots,\hat{\rho }_{k}). $$

Further, the double $\operatorname{AR}(p)$ model can yield the recursion formula

$$\varepsilon_{t}(\theta) = y_{t} - \sum _{i = 1}^{p} \phi_{i}y_{t - i}. $$

For $\Phi ( B ) = 1 - \phi_{1}B - \phi_{2}B^{2} -\cdots - \phi _{p}B^{p}$, where *B* is the back-shift operator, that is, $\varepsilon_{t} ( \theta ) = \Phi ( B ) y_{t}$. Then

$$\frac{\partial \varepsilon_{t} ( \theta ) }{\partial \theta } = \bigl( - \Phi^{ - 1} ( B ) \varepsilon_{t - 1} ( \theta ), - \Phi^{ - 1} ( B ) \varepsilon_{t - 2} ( \theta ), \ldots, - \Phi^{ - 1} ( B ) \varepsilon_{t - P} ( \theta ) \bigr) ^{\prime }. $$

Specially, let $\varepsilon_{t} ( \theta_{0} ) = \varepsilon _{t}$, the $\phi_{i}^{*}$ is coefficient of $\Phi^{ - 1} ( z ) = \sum_{i = 0}^{\infty } \phi_{i}^{*} z^{i}$, when $i < 0$, we have $\phi_{i}^{*} = 0$. From the above we can draw the following:

$$\frac{\partial \varepsilon_{t}}{\partial \theta } = \frac{\partial \varepsilon_{t} ( \theta ) }{\partial \theta } = \sum_{i = 1} ^{\infty } \varepsilon_{t - i} \lambda_{i}, $$

where $\lambda_{i} = ( - \phi_{i - 1}^{*}, - \phi_{i - 2}^{*},\ldots, - \phi_{i - p}^{*} ) ^{\prime } $.

#### Theorem 2

*If model* (1) *satisfies Assumptions* 1*–*3, *then it holds*

$$\sqrt{n} \hat{\rho } \to {}_{d}N ( 0,\Sigma ), $$

*where* $\hat{\rho } = ( \hat{\rho }_{1},\hat{\rho }_{2},\ldots, \hat{\rho }_{m} ) ^{\prime } $.

#### Proof

Let $\Lambda_{m} = ( \lambda_{1},\lambda_{2},\ldots, \lambda_{m} ) $. Then

$$\Lambda_{m}=\left(\begin{array}{cccccc} -1 & -\phi_{1}^{*} & \cdots & \cdots & \cdots & -\phi_{m-1}^{*}\\ 0 & -1 & \cdots & \cdots & \cdots & -\phi_{m-2}^{*}\\ \vdots & \vdots & \vdots & \vdots & \vdots & \vdots \\ 0 & 0 & \cdots & -\phi_{1}^{*} & \cdots & -\phi_{m-p}^{*} \end{array}\right)$$

is a $p \times m$ matrix, further

$$\Lambda_{\infty } \Lambda '_{\infty } = \sum _{i = 1}^{\infty } \lambda _{i} \lambda '_{i}. $$

Let $J ( l,l' ) = \sum_{h = - \infty }^{\infty } E ( \varepsilon_{t}\varepsilon_{t + l}\varepsilon_{t + h} \varepsilon_{t + h + l'} ) $. Then we obtain

$$H_{i,j} = H \bigl( l,l' \bigr) _{1 \le l \le i, \le l' \le j} = \frac{1}{ \sigma^{4}}J \bigl( l,l' \bigr) _{1 \le l \le i, \le l' \le j},\quad i,j = 1, \ldots, \infty. $$

According to Theorem 2 of Francq et al. [11], it holds that

$$\begin{aligned} \Sigma =& H_{m,m} + \Lambda '_{m} \bigl\{ \Lambda_{\infty } \Lambda '_{ \infty } \bigr\} ^{ - 1}\Lambda_{\infty } H_{\infty,\infty } \Lambda '_{\infty } \bigl\{ \Lambda_{\infty } \Lambda '_{\infty } \bigr\} ^{ - 1}\Lambda_{m} \\ &{} - \Lambda '_{m} \bigl\{ \Lambda_{\infty } \Lambda '_{ \infty } \bigr\} ^{ - 1}\Lambda_{\infty } H_{\infty,m} \\ &{} - H_{\infty,m}\Lambda '_{\infty } \bigl\{ \Lambda_{\infty } \Lambda '_{\infty } \bigr\} ^{ - 1}\Lambda_{m}. \end{aligned}$$

Now, we will present the limit distribution of the autocorrelation function of the residuals. It follows from Theorem 1 of Francq et al. [11] that

$$\begin{aligned} \operatorname{cov}( \sqrt{n} \hat{\zeta }_{l},\sqrt{n} \hat{\zeta }_{l'} ) =& \frac{1}{n}\sum_{t = 1}^{n - l} \sum_{t' = 1}^{n - l'} E ( \varepsilon_{t} \varepsilon_{t + l}\varepsilon_{t'} \varepsilon_{t' + l'} ) \\ \to& J \bigl( l,l' \bigr) \quad \text{as } n \to \infty \end{aligned}$$

When $p > 0$, let $\hat{\varepsilon } = \varepsilon_{t} ( \hat{\theta } ) $ approximately equal $e_{t}(\hat{\theta } )$. According to the above conditions, we have

$$\begin{aligned} \hat{\zeta }_{k} =& \frac{1}{n}\sum _{t = 1}^{n - k} \hat{\varepsilon } _{t}\hat{ \varepsilon }_{t + k} \approx \frac{1}{n}\sum _{t = 1}^{n - k} e_{t}(\hat{\theta } )e_{t + k}(\hat{\theta } ) \\ =& \frac{1}{n}\sum_{t = 1}^{n - k} \bigl[ e_{t}(\hat{\theta } )e_{t + k}( \hat{\theta } ) - \hat{ \varepsilon }_{t}(\hat{\theta } ) \hat{\varepsilon }_{t + k}( \hat{\theta } ) \bigr] + \frac{1}{n} \sum_{t = 1}^{n - k} \hat{\varepsilon }_{t}(\hat{\theta } ) \hat{\varepsilon }_{t + k}(\hat{\theta } ) \\ \approx& R_{n} + \frac{1}{n}\sum_{t = 1}^{n - k} \varepsilon_{t}(\theta _{0})\varepsilon_{t + k}( \theta_{0}) + \frac{1}{n}\sum_{t = 1}^{n - k} \biggl[ \varepsilon_{t + k}(\theta_{0})\frac{\partial \varepsilon_{t}( \theta_{0})}{\partial \hat{\theta }} + \varepsilon_{t}(\theta_{0})\frac{ \varepsilon_{t + k}(\theta_{0})}{\partial \hat{\theta }} \biggr] (\hat{ \theta } - \theta_{0}) \\ =& R_{n} + \zeta_{k} + \frac{1}{n}\sum _{t = 1}^{n - k} \biggl[ \varepsilon_{t + k} \frac{\partial \varepsilon_{t}}{\partial \theta '} + \varepsilon_{t}\frac{\partial \varepsilon_{t + k}}{\partial \theta '} \biggr] (\hat{ \theta } - \theta_{0}) \\ \approx& R_{n} + \zeta_{k} + E \biggl( \varepsilon_{t}\frac{\partial \varepsilon_{t + k}}{\partial \theta '} \biggr) (\hat{\theta } - \theta_{0}) \\ =& R_{n} + \zeta_{k} + \sigma^{2} \lambda_{k}'(\hat{\theta } - \theta_{0}), \end{aligned}$$

where

$$R_{n} = \frac{1}{n}\sum_{t = 1}^{n - k} \bigl[ e_{t}(\hat{\theta } )e _{t + k}(\hat{\theta } ) - \hat{ \varepsilon }_{t}(\hat{\theta } ) \hat{\varepsilon }_{t + k}( \hat{\theta } ) \bigr] . $$

So there exist constants $K > 0$ and $\rho \in ( 0,1 ) $ such that the following inequalities

$$\begin{aligned}& \sup_{\theta \in \Theta } \bigl\vert e_{t}(\hat{\theta } )e_{t + k}( \hat{\theta } ) - \hat{\varepsilon }_{t}(\hat{ \theta } ) \hat{\varepsilon }_{t + k}(\hat{\theta } ) \bigl\vert \le K\rho^{t}, \\& R_{n} \le \frac{1}{n}\sum_{t = 1}^{n - k} K\rho^{t} \le \frac{1}{n} = O_{p}\biggl( \frac{1}{n}\biggr) \end{aligned}$$

hold. Therefore,

$$\hat{\boldsymbol{\zeta }}_{k} = \boldsymbol{\zeta }_{k} + \sigma^{2} \Lambda_{k}'(\hat{\theta } - \theta_{0})+ O_{p}\biggl( \frac{1}{n}\biggr). $$

In the end, it holds that

$$\begin{aligned}& n \biggl( \frac{\hat{\zeta }_{k}}{\hat{\zeta }_{0}} - \frac{ \hat{\zeta }_{k}}{\sigma^{2}} \biggr) = \sqrt{n} \hat{\zeta }_{k}\frac{ \sqrt{n} ( \sigma^{2} - \hat{\zeta }_{0} ) }{\sigma^{2} \hat{\zeta }_{0}} \\& \quad \Rightarrow \quad \hat{\boldsymbol{\rho }}_{k} = \frac{ \hat{\boldsymbol{\zeta }}_{k}}{\sigma^{2}} + O_{p} \biggl( \frac{1}{n} \biggr). \end{aligned}$$

The proof of Theorem 2 is finished. □

In financial applications, it is often necessary to test whether some of the autocorrelation functions of the residual are zero at the same time. Box and Pierce (1970) proposed a portmanteau test statistic

$$Q_{m} = n\sum_{k = 1}^{m} \hat{ \rho }_{k}^{2} $$

to test

$$H_{0} = \rho_{1} = \rho_{2} = \cdots = \rho_{m} = 0 $$

or

$$H_{1}:\exists i \in \{ \boldsymbol{1},\boldsymbol{2},\ldots, \boldsymbol{m} \} ,\quad \rho_{i} \ne 0. $$

Ljung and Box (1978) modified the Q(m) statistic as

$$\bar{Q}_{m} = n ( n + 2 ) \sum_{k = 1}^{m} \frac{\hat{\rho } _{k}^{2}}{n - k} $$

to increase the power of the test in finite samples.

The decision rule is to reject $H_{0}$ if $\bar{Q}_{m} > \chi_{\alpha }^{2}$, where $\chi_{\alpha }^{2}$ denotes the $100 ( 1 - \alpha ) $ percentile of a chi-squared distribution with *m* degrees of freedom.

According to Theorem 2, we can directly get the exact asymptotic distribution of the portmanteau statistics.

#### Theorem 3

*It is true that*

$$\bar{Q}_{m} \to_{d}\sum_{i = 1}^{m} \bar{\xi }_{mi} z_{i}^{2}, $$

*where* $z_{1},z_{2},\ldots,z_{m}$ *is independent* $N ( 0,1 ) $ *random variables and* $\tilde{\xi }_{m} = ( \tilde{\xi }_{m1}, \tilde{\xi }_{m2},\ldots,\tilde{\xi }_{mm} ) ^{\prime } $ *is an eigenvector of* Σ.

It follows from Theorem 3 that $\bar{Q}_{m}$ is always a portmanteau test statistic of residual autocorrelation function under the condition of quasi-maximum exponential likelihood estimator. The conclusion is derived that the double $\operatorname{AR}(p)$ model with the quasi-maximum exponential likelihood estimator can be used to test the diagnostic results of the portmanteau test statistic.

## An empirical study

To study the law of financial market development, researchers generally select some indices to investigate the features of comprehensive economics and reflect the overall rather than one-sided trend of economic development in order to ensure the conclusions proposed appropriately for most phenomena. The CSI 300 index is one of indexes with these characteristics which can reflect the situation of Chinese stock market.

This article selects recent closing price data of CSI 300 index (399300) from December 1, 2016 to March 16, 2018, 315 sample observations in total. We used statistical software MATLAB to conduct research and analysis. The data can be downloaded from *Netease Finance*.

It is shown in Table 1 that the skewness is −1.1129, the return series is left skewed and the sequence distribution obtained is asymmetric. The kurtosis is $7.5884>3$, and the sequence presented has a high peak. The critical value under the 0.05 significance of the JB statistic is $5.9915<340.2778$. As is shown, the assumption of a normal distribution is not true, and this return series is heavy-tailed distribution.

**Table 1:** Main digital feature of CSI 300 index

Mean	Std.	Skewness	Kurtosis	Minimum	Maximum	JB statistic
0.0004	0.0077	−1.1129	7.5885	−0.0437	0.0214	340.2778

From Table 2, we can see that the value of *t* statistic of ADF test and PP test, respectively, is less than the critical value in the significance level in 1%, 5%, and 10%, and *p* value of *t* statistic approaches zero. Hence, we can judge the rejection of the original hypothesis. Then we can draw the conclusion that the sequence is a stationary sequence.

**Table 2:** Stationary test table

Test method	1% critical value	5% critical value	10% critical value	*t*-statistic	Probability
ADF test	−3.451078	−2.870561	−2.571647	−16.06732	0
PP test	−3.451078	−2.870561	−2.571647	−16.07588	0

We can see from Table 3 that there is no obvious difference between *p* values. Since *Q* statistic is zero, it shows that the original hypothesis does not hold in the significance of 5%. And then the return sequence obtained has relevance. For the observation data at different time, the corresponding variance is also different. So, it is of practical significance to test whether the sequence has heteroscedasticity.

**Table 3:** The autocorrelation function value and the partial autocorrelation function value of the sequence

Lag	ACF	PACF	Q-Statistic	P
1	−0.463	−0.463	67.786	0.0000
2	−0.068	−0.359	69.232	0.0000
3	0.172	−0.054	78.664	0.0000
4	−0.18	−0.175	88.993	0.0000
5	−0.036	−0.253	89.407	0.0000
6	0.11	−0.158	93.264	0.0000
7	−0.087	−0.183	95.725	0.0000
8	0.039	−0.146	96.224	0.0000
9	0.032	−0.138	96.555	0.0000
10	0.028	−0.024	96.803	0.0000
11	0.036	0.092	97.237	0.0000
12	−0.163	−0.126	105.99	0.0000
13	0.104	−0.057	109.51	0.0000
14	−0.004	−0.014	109.52	0.0000

**Figure 1:**
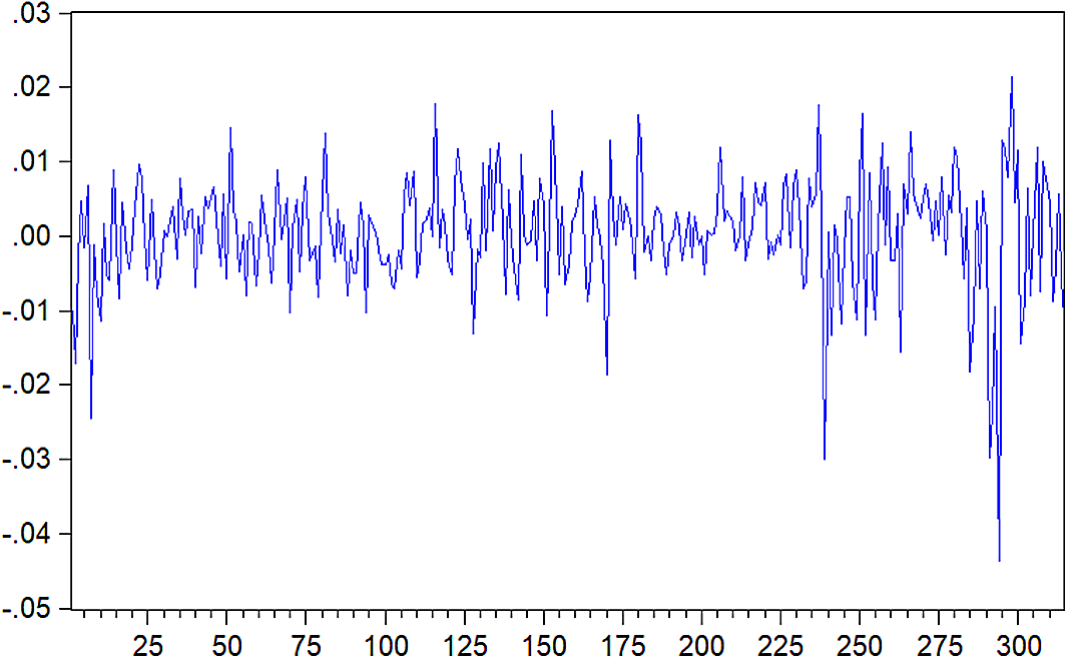
Residual sequence plot

From Fig. 1, residual has less fluctuation in November 2017 to February 2018, and fluctuates greatly in January 2017 to April 2017. This shows that conditional heteroscedasticity may exist in the presence of residual. Here, we use square residuals to analyze heteroscedasticity of the residual sequence. The square residuals can be expressed as follows:

$$\hat{\eta }_{t}^{2} = \frac{\hat{\varepsilon }_{t}^{2}}{\sqrt{ \hat{h}_{t}^{2}}}. $$

**Figure 2:**
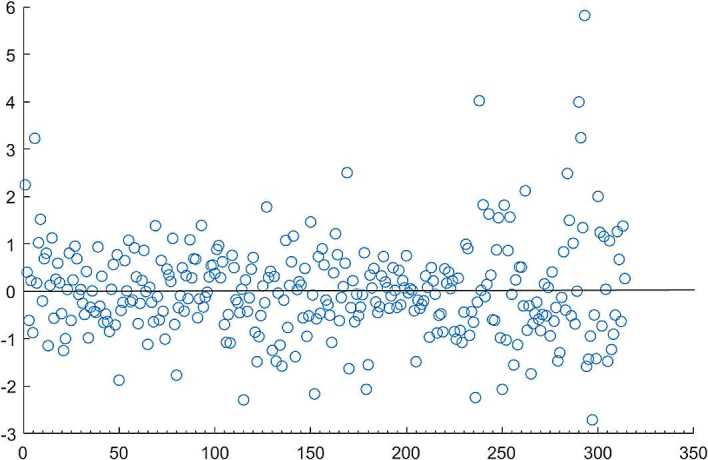
Heteroscedasticity test scatter plot

In Fig. 2 most of the scattered points deviate from the mean, showing that the unspecific shape is around the average. Obviously, the residual sequence has heteroscedasticity.

In order to verify the usefulness of the double $\operatorname{AR}(p)$ model under autocorrelation function, it is necessary to estimate the parameters for the given model under the actual data. The effect of model fitting has a direct impact on the accuracy of data prediction. Thus, model parameter estimation must be carried out firstly.

Regarding the approaches of parameter estimation, scholars put forward many methods of estimation for model parameters, such as moment estimation, least squares estimation, maximum likelihood estimation, and so on. In application, however, the quasi-maximum exponential likelihood estimator is used widely because of its excellent properties. Thus, we use quasi-maximum exponential likelihood estimator to make parameter estimation in this part.

Since the ARCH model, proposed by Engle [21], has many advantages, the ARCH model has been widely used in the simulation of economic and financial data. Later, the ARCH model has been extended to the GARCH model. The innovation of GARCH model can be rewritten as an ARMA form

$$\varepsilon_{t}^{2} = \alpha_{0} + \sum _{i = 1}^{\max ( p,q ) } ( \alpha_{i} + \beta_{i} ) \varepsilon_{t - 1} ^{2} + \tau_{t} - \sum_{j = 1}^{q} \beta_{j}\tau_{t - j}, $$

where $\tau_{t} = \varepsilon_{t}^{2} - \sigma_{t}^{2}$.

If the AR polynomial in the GARCH model has a unit root, then we can obtain the IGARCH model. Because the IGARCH model has a similar ARCH effect to the double $\operatorname{AR}(p)$ model, through some statistic of IGARCH model obtained we can compare the effect of portmanteau test statistic for the double $\operatorname{AR}(p)$ model. Then we take $\operatorname{IGARCH}(1, 1)$ and double $\operatorname{AR}(1)$ model as a group example. The $\operatorname{IGARCH}(1, 1)$ model is as follows:

$$\begin{aligned}& \varepsilon_{t}^{2} = \eta_{t}^{2}h_{t}, \\& \sigma_{t}^{2} = \alpha_{0} + \beta_{1}\sigma_{t - 1}^{2} + ( 1 - \beta_{1} ) \varepsilon_{t - 1}^{2}. \end{aligned}$$

On the basis of quasi-maximum exponential likelihood estimation, this paper uses the nonlinear multivariate function to determine the initial value. Finally, we can obtain model parameter estimation: $\alpha_{0} = 0.5093$, $\beta_{1} = 0.2501$. For $\beta_{1}$ reflects the correlation between the observed data. This data indicates that the CSI 300 index has a weak sequence correlation in this period. The results show that the volatility of the CSI 300 index has a short duration.

Finally, as is shown in Sect. 2, it is necessary to do a portmanteau test of the double $\operatorname{AR}(1)$ model, which is the core of this section. The test statistic of $\operatorname{IGARCH}(1, 1)$ gradually obeys the $\chi^{2}$ distribution. When the lag $n = 7$, and the significance level is 0.05, we can draw that $\chi^{2} ( 7 ) = 14.067$, $\chi^{2} ( 14 ) = 23.685$. When the lag $n = 14$, we can get that $\chi^{2} ( 28 ) = 41.337$. The results of the portmanteau test for double $\operatorname{AR}(1)$ model are presented in Table 4.

**Table 4:** Simulation results of portmanteau test statistic

Statistic	Lag *n* = 7	Lag *n* = 14
$\bar{Q}_{m}$	5.376	22.896

From Table 4, it is obvious that no matter the lag is 7 or 14, the portmanteau test statistic $\bar{Q}_{m}$ is always less than the $\chi^{2}$ statistic with different degrees of freedom in the same lag. Therefore, we can judge that the $\operatorname{AR}(1)$ model is tested by portmanteau test based on the quasi-maximum exponential likelihood, and thus, the model fitting is reasonable.

## Conclusions

This paper proposes the quasi-maximum exponential likelihood estimator and constructs the portmanteau test for the double $\operatorname{AR}(p)$ model of residual autocorrelation function based on certain assumptions. We select a part of the history data of the CSI 300 index closing price data to make an empirical study for the double $\operatorname{AR}(p)$ model. The conclusions are as follows:

(i) The CSI 300 index return sequence has weak correlation in the selected time period, with a short duration and no long memory.
(ii) On the basis of quasi-maximum exponential likelihood estimation method, the double $\operatorname{AR}(p)$ model is fitted. Then a diagnostic test for this model is conducted by using portmanteau test statistic based on residual partial autocorrelation function. It is concluded that the double $\operatorname{AR}(p)$ model is reasonable in practical application.
